# Detection of vertebral fracture in an acute hospital setting: an intervention to reduce future fracture risk through fracture liaison service intervention?

**DOI:** 10.1007/s11657-020-00832-2

**Published:** 2020-10-10

**Authors:** Michael Toal, Connor McLoughlin, Nicole Pierce, Julie Moss, Sarah English, John R Lindsay

**Affiliations:** 1 Mater Infirmorum Hospital, Belfast Health & Social Care Trust, Belfast, Northern Ireland; 2 Royal Victoria Hospital, Belfast Health & Social Care Trust, Belfast, Northern Ireland; 3 Musgrave Park Hospital, Belfast Health & Social Care Trust, Belfast, Northern Ireland

**Keywords:** Vertebral, Fracture, Osteoporosis, Identification

## Abstract

***Summary*:**

We introduced a standardised reporting system in the radiology department to highlight vertebral fractures and to signpost fracture prevention services. Our quality improvement project achieved improved fracture reporting, access to the FLS service, bone density assessment and anti-fracture treatment.

**Purpose:**

Identification of vertebral fragility fractures (VF) provides an opportunity to identify individuals at high risk who might benefit from secondary fracture prevention. We sought to standardise VF reporting and to signpost fracture prevention services. Our aim was to improve rates of VF detection and access to our fracture liaison service (FLS).

**Methods:**

We introduced a standardised reporting tool within the radiology department to flag VFs with signposting for referral for bone densitometry (DXA) and osteoporosis assessment in line with Royal Osteoporosis Society guidelines. We monitored uptake of VF reporting during a quality improvement phase and case identification within the FLS service.

**Results:**

Recruitment of individuals with VF to the FLS service increased from a baseline of 63 cases in 2017 (6%) to 95 (8%) in 2018 and 157 (8%) in 2019 and to 102 (12%) in the first 6 months of 2020 (*p* = 0.001). One hundred fifty-three patients with VFs were identified during the QI period (56 males; 97 females). Use of the terminology ‘fracture’ increased to 100% (mean age 70 years; SD 13) in computed tomography (*n* = 110), plain X-ray (*n* = 37) or magnetic resonance imaging (*n* = 6) reports within the cohort. Signposting to DXA and osteoporosis assessment was included in all reports (100%). DXA was arranged for 103/153; 12 failed to attend. Diagnostic categories were osteoporosis (31%), osteopenia (36%) or normal bone density (33%). A new prescription for bone protection therapy was issued in 63/153. Twelve of the series died during follow-up.

**Conclusions:**

Standardisation of radiology reporting systems facilitates reporting of prevalent vertebral fractures and supports secondary fracture prevention strategies.

## Introduction

Vertebral fractures are the most common osteoporosis fracture and are associated with significant morbidity [1]. While the presence of incident vertebral fracture predicts future vertebral and hip fractures, a lack of awareness of the clinical significance of vertebral fracture events exists. Under-diagnosis of vertebral fractures is common in clinical practice [1]. Lack of recognition by patients of back pain symptoms arising from vertebral fracture and by healthcare providers due to variability in reporting terminology for fractures or access to imaging may contribute. Finally, the referring clinician may not be aware of, or seek access to, fracture liaison services [2].

The prevalence of vertebral fractures has been reported at around 12% in women aged 50–79 years and up to 20% women over 80 years [1]. A majority of these will be osteoporotic in nature [1]. Identification, diagnosis, and treatment of osteoporosis following a vertebral fragility fracture provide an opportunity to identify individuals with osteoporosis who might benefit from fracture prevention strategies.

We undertook a quality improvement project (QIP) within radiology, to raise awareness of fracture prevention strategies. Our goal was to integrate vertebral fracture identification within diagnostic imaging departments in order to actively seek vertebral fractures apparent on any spine imaging and to report vertebral fractures clearly and unambiguously [1]. A voice prompt reporting system was introduced within our picture archiving and communication system (PACS), to flag the presence of vertebral fracture and to signpost referring clinicians to fracture prevention services according to Royal Osteoporosis Society guidelines [1]. We sought to promote alerting the referring clinician to the need for further assessment of fracture risk, via the FLS [2].

## Methods

Our service improvement programme was undertaken within the radiology departments of the Mater Infirmorum and Musgrave Park Hospitals, Belfast Health & Social Care Trust, to raise awareness of vertebral fracture identification and management, in line with the Royal Osteoporosis Society (ROS) guideline “Clinical Guidance for the Effective Identification of Vertebral Fractures” [1]. We initially examined the prevalence of vertebral fractures in a baseline retrospective study of 154 non-traumatic CT imaging scans and radiology reports from Sectra Radiology Imaging Services (RIS), in those aged 50 years or older. Imaging and reports were assessed for fracture identification and terminology, compared with standards based on ROS guidelines [1].

A standard operating procedure (SOP) was then agreed between the fracture liaison service (FLS)/osteoporosis team and radiology departments for vertebral fracture identification and reporting arrangements for plain X-ray, computed tomography (CT) and magnetic resonance imaging (MRI) as part of a new Vertebral fracture pathway (Appendix 1). We provided educational sessions on VF identification and fracture prevention. The QIP was included within departmental audit programmes to agree to use the term ‘fracture’, using the Genant semi-quantitative method, in reports when a vertebral fracture is present, and to avoid a range of terminology used that may not necessarily feature the word fracture [1, 3]. Genant vertebral fracture staging charts were provided at radiology stations [3]. Our SOP highlighted the need for routine sagittal reformatting of CT/MRI images using bone algorithms, either by the operator or by the reporting clinician with scrutiny of lateral views of the spine on any relevant images. We introduced a voice command, using speech magic to insert a pre-defined text within a report when an incidental finding of grade 2 and 3 vertebral fragility fractures was discovered [4]. A team of consultant radiologists were asked to insert the defined voice command ‘PROMPT VFP’ into reports where there were findings of vertebral fracture as follows: ‘Appearances suggest a high risk of fragility fracture and referral for DXA Scan and Osteoporosis assessment is advised’. These systems were designed to support fail-safe alert mechanism in respect of vertebral fractures as ‘significant, important, unexpected and actionable findings’ in accordance with the Royal College of Radiology (RCR) standards [1, 5]. The clinical lead for the radiology service (NP) was asked to scrutinise a selection of the imaging reports within the department for quality assurance purposes using the same methods for the baseline audit.

We hypothesised that introduction of our reporting system and QIP would improve rates of vertebral fracture reporting, signposting of the need for assessment of osteoporosis and in turn increase rates of recruitment to our FLS service.

**Fig. 1:**
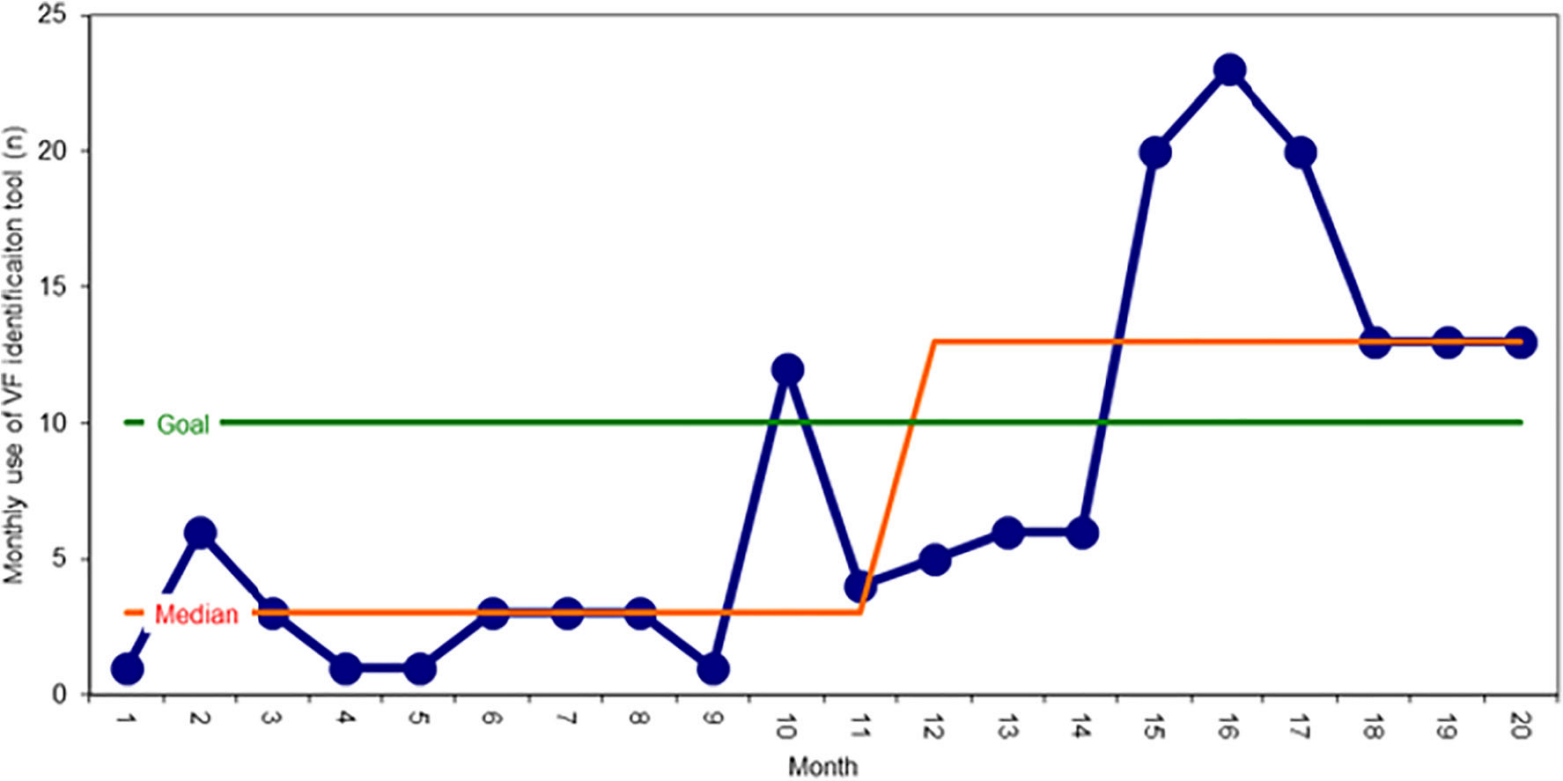
Run chart of frequency of uptake of the vertebral fracture (VF) identification reporting tool. The median of the data is presented in red and ranged 3–13 cases per month. VF reporting increased significantly from a baseline of 1–6 cases per month in the first 9 months of the QI programme. The target of 10 cases per month is shown as a horizontal green line and was achieved consistently in the last 6 months of the analysis period

The primary drivers for the QIP were to ensure unambiguous reporting of vertebral fractures, signposting of the need for FLS assessment, and to develop a process for patient flow to the FLS service. Secondary drivers were to improve awareness of the clinical importance of vertebral fractures within radiology, a system to flag newly identified fractures, and to improve communication and linkages between radiology and the FLS team. A team comprised of an osteoporosis physician, radiologist, radiology administrator and fracture liaison nurse contributed to the design and delivery of the project. The process for VF identification and reporting was agreed at a series of radiology audit meetings attended by the various stake holders. The service improvement plan broadly followed plan, do, study, act (PDSA) cycles with use of run charts to monitor use of the VF reporting tool on a monthly basis (Fig. 1). The programme was introduced on a pilot basis being aware of the potential risks and benefits of increasing referral volumes to the FLS service (Table 1). The clinical lead for the team circulated the monthly run chart to the radiology teams to feedback on progress with the QI project with VF reporting, to support user engagement and to monitor sustainability across the radiology and FLS teams. The Health Trust is comprised of 3 acute hospitals and one elective site. The majority (76%) of our QI series scans were undertaken at the Mater Infirmorum Hospital, a small acute hospital, which undertakes around 17,895 cross-sectional imaging scans annually (CT, *n* = 10,941; MR, *n* = 6954). The other 24% of scans were undertaken at Musgrave Park Hospital, the base within the Trust for the osteoporosis and orthopaedic specialist service.

**Table 1:** Quality improvement plan methodology-risks and benefits of introduction of VF reporting system including balancing measures

Risks associated with VF reporting system	Balancing measures	Comments
Fractures inappropriately attributed to osteoporosis	Access to DXA/secondary screen for osteoporosis to support diagnostic process	FLS service are well placed to review clinical presentation
Alternative diagnoses Myeloma Other malignant process	Access to screening for secondary causes of osteoporosis Radiology reporting provides support for diagnostic process	High-risk patients will access assessment Individuals with established vertebral fractures will access assessment/treatment
Distinguishing new acute fractures from old VFs	FLS recruitment processes are well established	Risk of overloading DXA unit with high volume of referrals Under-detection of VFs is well recognised

A prospective database of vertebral fracture cases was maintained by data extraction from Sectra RIS using a search function for VF reports. Data on demographics, gender, age, vertebral fracture location, co-morbidities, mortality data, bone protection prescriptions and clinical outcome at last date of follow-up were obtained from the electronic clinical record using a number of regional Electronic Records systems, (Orion Health – Concerto; Sectra – PACS Workstation IDS7). Initial assessment approaches were directed at determining whether the referring clinician responded to the reporting arrangements through referral to direct access DXA services or osteoporosis clinics. It became apparent that active in reach and pull-out by the FLS service would be needed to optimise patient identification and recruitment. Patients were invited to face-to-face FLS clinics and received a patient information leaflet in advance of clinic highlighting the role of FLS in fracture identification and fracture prevention. A text reminder service is in place to remind patients of planned appointment times.

Assessment of bone mineral density (BMD) readings was undertaken with the GE Lunar iDXA scanner, which has a reported least significant change of 0.033 g/cm^2^. World Health Organization (WHO) diagnostic criteria for osteoporosis were used [6, 7]. DXA scanning was undertaken by a trained radiographer within a musculoskeletal imaging department and reviewed by the FLS team comprised of an FLS nurse and physician, who is certified by the International Society for Clinical Densitometry.

The Belfast FLS service provides trauma services for the greater Belfast Area and wider region. Our estimated case load from the national hip fracture database is 451 hip fractures per year with an estimated fragility fracture incidence of 2225 annually. Based on the hip fracture incidence, we estimate the prevalence of clinical and radiological VFs of 338 for clinical and 676 VFs, respectively, based on expert consensus [8]. This pilot was introduced at the two smaller hospitals within the healthcare Trust on a pragmatic basis for operational reasons and to test the practicalities of the reporting arrangements.

### Outcomes

Outcomes of VF reporting were measured based upon monthly PACS reports where the VF identification tool was used. We measured FLS recruitment rates for VF fracture patients on a monthly basis. Clinical outcomes including DXA imaging and anti-osteoporosis treatment use were recorded on the Northern Ireland electronic healthcare record (NIECR). Our primary outcome was to determine the effectiveness and use of the radiology reporting tool to optimise VF reporting and recruitment to DXA and FLS services. The QIP continued until we exceeded 150 patients and with stable case identification over a 3-month period as shown in the run chart (Fig. 1.)

### Statistical methods

We examined clinical demographics including age, gender, fracture location and co-morbidities. We measured uptake of the reporting tool on a monthly basis using run chart methodology (Fig. 1.). All results were analysed using Microsoft Excel 2016. Continuous data was presented as mean, standard deviation and range. Chi-squared testing was used to determine where there was any difference between the observed and the expected values. Unpaired *t* tests were used to compare age ranges due to unequal variances. ANOVA was used for multiple comparisons. Results were considered significant if the *p* value was < 0.05.

## Results

The prevalence of incidental moderate to severe vertebral body fractures in our baseline audit prior to introduction of the VF reporting tool was 24/154 (15.6%) [13 females/11 males; mean age 75 years, SD 11 years]. 13/24 had a pre-existing history of osteopenia or osteoporosis. Pre-existing vertebral body fractures were noted in 11/24 (Table 2). The most common vertebral fracture location was L1. Multiple fractures were noted in 11/24. 20/24 of moderate-severe vertebral body fractures were identified during the audit but were reported using a wide variation in terminology including compression (*n* = 4), height loss (*n* = 1), and wedging (*n* = 2), whereas the term fracture was used in only 13/24 (54%).

**Table 2:** Study population and demographics of baseline retrospective cohort

Age [years (SD)]	69 [11.3]
Gender	56 Male; 97 Female
Age < 75 years (%)	109 (71)
Imaging modality; *n* (%)	
CT	154 (100)
CT type	
Abdomen and pelvis	56
Abdomen non-contrast	1
Chest abdomen and pelvis	30
Chest with contrast	23
CT KUB/kidneys	18
CTPA	26
Vertebral fracture identified (%)	24/154 (15.6)
Single VF	11
Multiple VFs	13
Pre-existing known fracture (%)	11/24 (45.8)

**Fig. 2:**
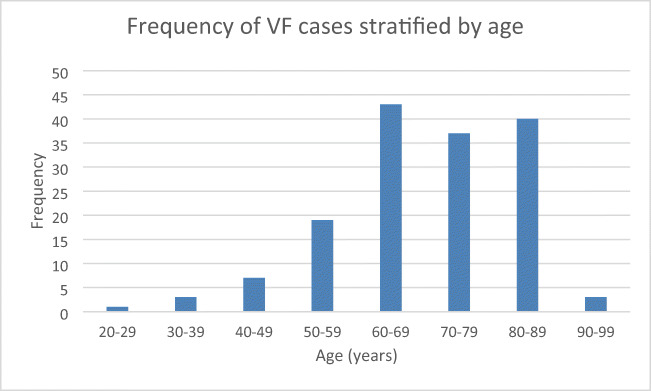
Frequency of moderate to severe vertebral fracture stratified by age

During implementation of vertebral fracture identification QIP, the VF tool was subsequently used in reports for 153 patients with vertebral fractures (56 males; 97 females). In 100% of these cases, the terminology ‘fracture’ was used, which is a significant improvement from baseline (*p* < 0.05), meeting the ROS audit standards for vertebral fracture identification guidance (Table 3) [1]. The mean age was 70 years (SD 13 years), which was similar to the baseline audit series (*p* = 0.16) and was negatively skewed (Fig. 2.). The QIP series included a higher proportion of females at 63% compared with the baseline audit at 49% (*p* = 0.01). Individuals were under the care of a range of specialist services (*n* = 12), most commonly with respiratory medicine (34%), emergency medicine (28%) and general surgery (9%). Twelve patients died during timeframe of the service improvement plan.

**Table 3:** Study population and demographics of prospective QIP series

Age at fracture diagnosis [years (SD)]	70 [13]
Age > 75 years (%)	65 (42.8%)
Gender	56 male; 97 female
Imaging modality; *n* (%)	
CT	110 (71.9)
MRI	6 (3.9)
Plain imaging	37 (24.2)
Newly identified fracture (%)	100 (65.4)
Prior known Osteoporosis (%)	29 (19.0)
IOF FLS indicators	
Proportion of estimated VF case load	
Time to DXA in under 75 years	16.9 weeks (3–72)
% with treatment initiation within 16 weeks	Unknown
DXA requested (%)	101 (66)
Age < 75 years (%)	65 (64)
Age > 75 years (%)	36 (36)
Prior DXA within 2 years (%)	19 (12.4)
Co-morbidities *n* (%)	Chronic obstructive pulmonary disease: 40 (26.1) Previous fracture: 35 (22.9) Hypertension: 27 (17.6) Diabetes mellitus: 25 (16.3) Malignancy: 21 (13.7) Ischaemic heart disease: 16 (10.5) Alcohol excess 14 (9.1) Hypothyroidism: 10 (6.5) Cognitive impairment: 8 (5) Stroke: 6 (4)

**Fig. 3:**
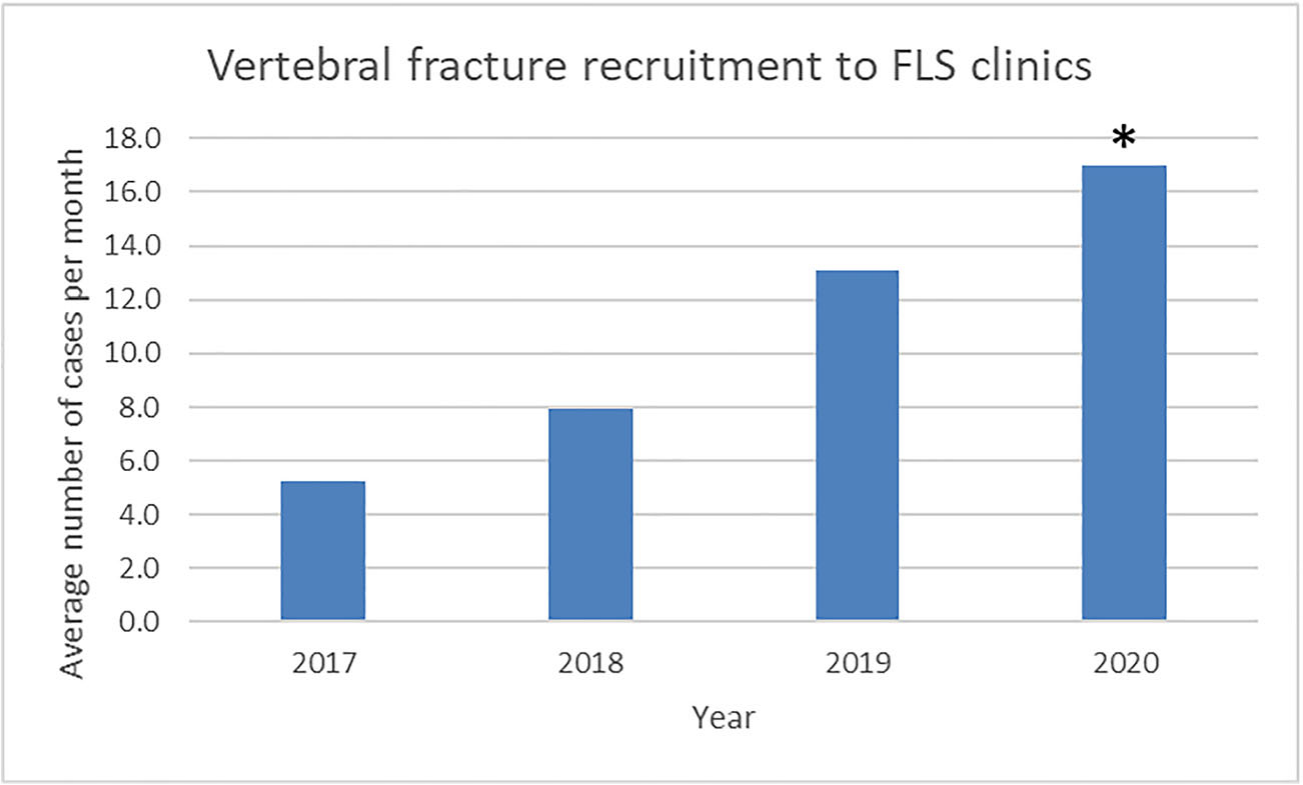
Frequency of vertebral fracture patients attending fracture liaison service clinics per month prior to and following QIP across the years 2017–2020 (*p = 0.02, ANOVA)

Within the last 12 months of the VF QI initiative, we identified 136 vertebral fractures (Fig. 1.). Recruitment of individuals with vertebral fracture to our FLS service increased from a baseline of 63 cases in 2017 (6%) to 95 (8%) in 2018 and 175 (9%) in 2019 and to 102 (12%) in the first 6 months of 2020 (Fig. 3.). The observed proportion of vertebral fractures was significantly higher in 2020 compared with baseline during the implementation of our QIP (*p* = 0.001). The mean time to assessment varied between 3 and 72 weeks from inception of the VF reporting tool with a mean time to assessment at the FLS service at 16.9 weeks for the cohort.

**Fig. 4:**
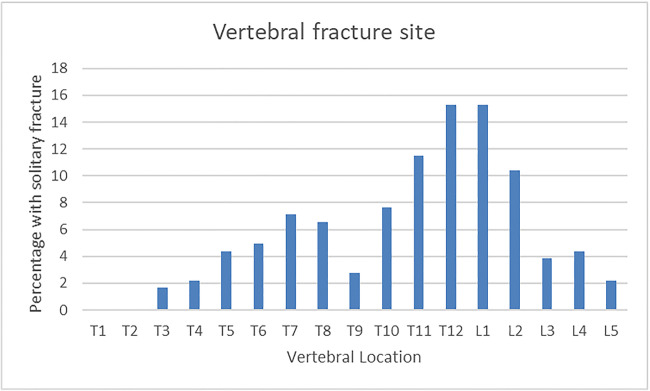
Distribution of individuals with solitary vertebral fractures by location across thoracic and lumbar spine sites

Monthly reporting of the VF reporting tool use identified that the tool was most commonly used during assessment with computed tomography (CT, *n* = 110 (72%) followed by plain X-ray *n* = 37 (24%) and magnetic resonance imaging (MRI) (*n* = 6 (4%)). A majority of vertebral fracture events were newly identified (*n* = 100; 65%), whereas the remainder (*n* = 53; 35%) were previously diagnosed. Multiple fractures were common (89/153). A majority of fractures were observed within the thoracic region (*n* = 159) as compared with the lumbar region (*n* = 74). The most common site of solitary fractures was located at T11 (11%), T12 (15%) and L1 (15%) (Fig. 4.). 25/153 imaging reports were scrutinised by a second consultant radiology, as clinical lead for radiology service for quality assurance purposes. There was strong concordance with the reported findings (24/25).

Documented co-morbidities within the study population (Table 3) included chronic obstructive pulmonary disease in 40/153 (26%), hypertension in 27/153 (18%) and diabetes mellitus in 25/153 (16%).

**Fig. 5:**
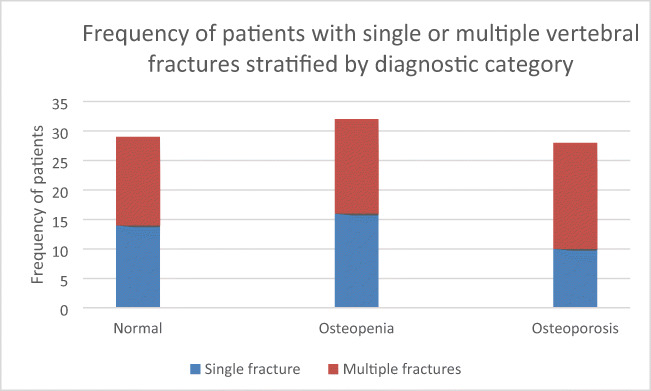
Frequency of patients with single or multiple vertebral fractures stratified by diagnostic category following DXA

The imaging report signposted clinicians to consider referral for DXA and osteoporosis assessment in all cases within the series (100%). Ninety-six of these were aged 50–80 years, who would normally fall within the recruitment age category for the local FLS service. A majority of the series had never had a formal assessment of bone health (104/153; 68%). 49/153 had previously undertaken DXA scanning; of these, 19/49 had attended for DXA within 2 years. A new referral for DXA was arranged for 101 of the series. Twelve cases failed to attend for DXA assessment following vertebral fracture identification. Of those who attended for DXA following VF identification 28/89 (31%) were diagnosed with osteoporosis, 32/89 (36%) with osteopenia and the remaining 29/89 (33%) had normal bone density according to WHO criteria. The proportion of individuals with a densitometric diagnosis of osteoporosis was higher at 18/34 for those with multiple vertebral fractures than for 10/26 for those identified with single vertebral fractures when stratified by T-scores criteria at DXA (*p* < 0.001, Fig. 5).

Within the study population, 29/153 had known osteoporosis. 55/153 were previously treated with calcium and/or vitamin D. 33/153 of the series were already receiving bisphosphonate treatment. A new prescription for bone protection therapy according to DXA criteria, with either calcium ± vitamin D and/or bisphosphonate, was issued following the report of a vertebral fracture in 63/153. In 12/63 of these treatment changes were issued without undertaking DXA.

## Discussion

We introduced a vertebral fracture identification pathway within radiology to optimise identification and reporting of VFs. We observed improved fracture reporting with signposting to the fracture liaison service in all cases, in line with recent quality standards, issued by the Royal Osteoporosis Society [2]. Of those attending for DXA, a third of cases had either osteoporosis or osteopenia on DXA. Around two-thirds were newly diagnosed, and a similar proportion had multiple fractures. Forty percent of the series had a new bone protection treatment initiated. Standardisation of radiology reporting systems facilitated reporting of prevalent VFs and secondary fracture prevention strategies.

VFs are the commonest form of osteoporotic fracture and can be viewed as a sentinel event. Population-based studies have identified the incidence of VF ranging from 100 to 800 per 100,000 person-years among those aged 65 years or older [9]. There is an increased risk of mortality of up to eight times compared with the age-matched population for individuals following a symptomatic VF, particularly within the first 6 months of the event [9]. Mortality rates are similar to those observed following hip fracture [10, 11]. The risks of loss of independence and mortality with VF are high and are associated with a significant risk of physical decline and institutionalisation [11].

Although there is some evidence to support population screening for osteoporosis targeted, case identification is the current standard of care in the UK [12]. In this context, VF identification represents an excellent opportunity to identify high-risk individuals with osteoporosis who may benefit from fracture prevention strategies. However, under-recognition of VFs is well recognised for both incidental and clinical fractures [1, 13]. Affected individuals may not seek medical attention as around two-thirds of individuals may be unaware of the presence of VF, ranging up to 82% being asymptomatic in one European series [14]. There is often a lack of recognition of the clinical significance of VFs by clinicians and radiologists leading to delayed diagnosis. Around a third to half of incidental radiographic VFs are not reported when present on imaging studies [15, 16]. A minority of cases, of around 12–23%, are diagnosed [1, 17–19], which may delay access to diagnostic and treatment interventions.

Failure to recognise the presence of VF events represents a lost opportunity to intervene with clinical interventions with proven anti-fracture efficacy. The recent Royal Osteoporosis Society guideline for VF identification is welcomed to improve awareness and systems for VF identification across the patient journey [1]. In our series, as illustrated by the run chart, the active pull out by our FLS service, local educational initiatives and participation in national audit programmes were noted to have a significant impact on VF identification and referral for DXA and attendance at FLS clinic (Figs. 1 and 3). Within the last 12 months of the VF QI initiative, we identified 136 morphometric vertebral fractures, which represent around 20% of the morphometric spine fractures that might be anticipated according to IOF key performance indicators for VF identification [8]. With the success of the recent pilot, we intend to extend the pilot across the two larger acute hospital sites within the Health Trust and would anticipate a significantly higher capture rate for VF detection with adoption of the VF reporting system.

The prevalence of VFs within Ireland was recently studied in a systematic review that highlighted that the true prevalence was difficult to ascertain due to definitions used and differences in the study populations [20]. McCabe et al. noted a VF prevalence ranging 5% among hospitalised population to 90% in older individuals with low bone density in whom a fracture was suspected [20]. Two prior studies from Belfast showed a prevalence of VF ranging from 2.7% in a fracture liaison service setting ranging up to 40.7% a cross-sectional study of a group of Northern Ireland men with low-trauma forearm fractures [21, 22]. More recently, in a UK national audit of vertebral fragility fractures in 6357 patients, a lack of compliance to ROS standards was noted with use of recommended terminology being achieved in 60.3%, a comment on fracture severity at 26.2% and recommendation for referral/further assessment in only 2.6% [1, 23]. Our baseline audit identified a prevalence of incidental moderate to severe VF in 15.6% of unselected consecutive series of 154 patients attending for computed tomography for investigation by a range of specialist services. A range of terminology was used to describe VFs, and in 46% of cases, the term fracture was not used. Following educational initiatives and roll out of the QIP, the term fracture was used in all cases of this series, which compares favourably to other UK settings [23]. It is difficult however to estimate the true prevalence of fractures within our wider Health Trust as these cases represent a small proportion of the total number of cases referred for imaging.

Osteoporosis management following VF remains inadequate when secondary fracture prevention programmes are not implemented [23]. Whereas epidemiological data in the USA show that VFs account for 27% of annual fragility fractures, UK data suggest that radiologists miss VFs in more than 50% of cases [23]. The important role of fracture liaison services, which are cost effective for fracture prevention, was highlighted in a recent US study [24]. In this series of retrospective series of 2933 patients attending an emergency department, 98% did not receive a DXA scan and only 7% were commenced on anti-resorptive therapy after their fracture. Thirty-eight percent of the series went on to develop a second fragility fracture within 2 years [24]. We observed steady uptake of DXA imaging in 67% of the cohort, supported by active pull-out of cases by our FLS team during the VF identification QIP.

In our series, 18% of cases had pre-existing osteoporosis. We were able to identify a significant number of new cases with undiagnosed osteoporosis or osteopenia using this method of case identification. While a proportion of cases had normal bone density on DXA, the majority had reduced bone mass in categories that might benefit from health promotion intervention and/or pharmacotherapy with calcium, vitamin D and or bisphosphonates. We observed a number of individuals who did not attend for DXA, and the importance of communicating the rationale for investigation and management is key. There is the possibility of overburdening the capacity for the fracture liaison team to offer appointments through wider scale implementation of the VF identification programme; however, at present, we have not yet encountered this issue. During the audit time frame, the proportion of VF cases among our FLS clinics increased from a baseline of 6% in 2017 to 12% in the first half of 2020; we would anticipate further progress as the VF identification programme rolls out across other hospitals within the health Trust [personal observation]. Progress will continue to be monitored as all health Trusts have enrolled in the UK Royal College of Physicians national FLS-database [25].

There are a number of limitations within our VF identification programme and analysis. Firstly, we are heavily dependent upon engagement of a wide range of reporting radiologists, among whom there was variable use of the reporting tool. Secondly, we were not able to accurately ascertain the true frequency of VF reporting, where the reporting tool was not used, and therefore case identification may be an underestimate. Thirdly, we recognise that grade 1 vertebral deformities with end plate disruption are recognised to confer future fracture risk and that the reported outcomes may represent and underestimate. Fourthly, as we limited the purpose of the study to examine the logistics of rolling out the ROS VF reporting systems, we need not systematically re-examine radiology images to explore the sensitivity and specificity of the QI initiative. Finally, our systems still rely on manual processes by the reporting radiologist and for VF searches and recruitment to DXA and FLS. These are critical steps in supporting access to fracture prevention services, and the use of the reporting tool will need to continue to be promoted. In the future, there may be opportunities to explore artificial intelligence solutions to support patient management.

In summary, based upon the existing literature and our experience, we support the recommendation for clinicians to work closely with diagnostic radiology departments and to introduce systems to improve VF reporting, in order to optimise vertebral fracture identification and fracture prevention strategies.

## Data Availability

Not applicable.

## Appendix. Vertebral fracture pathway – process within imaging services

Scheme of work to flag possible vertebral fracture within Sectra RIS

### Background

Vertebral fractures are the most common osteoporotic fracture. Vertebral fractures are most likely to be under-reported on imaging obtained for non-musculoskeletal indications. This includes images acquired using all modalities that involve any part of the thoracolumbar spine, with the greatest opportunity presented by the increasing number of CT scans undertaken in older adults.*

### Role of imaging in the vertebral fracture pathway

Whenever imaging that includes the spine is reported, it is anticipated that the report should indicate that the spine has been assessed. Furthermore, when a vertebral fracture has been identified the reporting radiologist should indicate within the report that the referring clinician should refer the patient for a DXA scan and osteoporotic assessment.*

It is proposed that a Standard Report Text is inserted to the report where there is suspicion of a vertebral fracture. The wording of the Report Text is below, and the recommendation is that radiologists to use the voice prompt function within Sectra RIS.

#### Standard Report Text

**Appearances suggest a high risk of fragility fracture and referral for DXA Scan and Osteoporosis assessment is advised.**

### User Guide to Inserting Text via Voice Prompt in Sectra RIS

- User must have the ‘Use Voice Commands’ box TICKED within the Dictation Window on RIS.
- Using speech magic voice recognition, say **‘PROMPT VFP’**.

If a user does not wish to use the voice prompts or is reporting without speech magic, then the text can be added from the ‘SELECT STANDARD REPORT’ ICON located above the report:

- From the pop up selection of Standard Reports, scroll to code ‘VFP’ and mouse click so text displays in box to the right.
- Click on ‘Select’.

*Reference from ‘Clinical Guidance for the Effective Identification of Vertebral Fractures’ National Osteoporosis Society: Publication Date: November 2017 Version 1

## References

[1] Clinical Guidance for the Effective Identification of Vertebral Fractures. Royal Osteoporosis Society. https://theros.org.uk/media/99101/vertebral-fractures-guidelines.pdf, last accessed 19/03/2020

[2] Effective Secondary Prevention of Fragility Fractures: Clinical Standards for Fracture Liaison Services https://theros.org.uk/media/1eubz33w/ros-clinical-standards-for-fracture-liaison-services-august-2019.pdf, last accessed 23/03/2020

[3] Genant HK, Wu CY, van Kuijk C (1993). Vertebral fracture assessment using a semiquantitative technique. J Bone Miner Res.

[4] Speech Magic: https://en.wikipedia.org/wiki/SpeechMagic, last accessed 30/3/20

[5] Standards for the communication of radiological reports and fail-safe alert notification. London: Royal College of Radiologists; 2016, p.6

[6] Kanis JA, Melton LJ, Christiansen C, Johnston CC, Khaltaev N (1994). The diagnosis of osteoporosis. J Bone Miner Res.

[7] Kanis JA, Adachi JD, Cooper C, Clark P, Cummings SR, Diaz-Curiel M, Harvey N, Hiligsmann M, Papaioannou A, Pierroz D, Silverman SL, Szulc P, and the Epidemiology and Quality of Life Working Group of IOF. Standardising the descriptive epidemiology of osteoporosis: recommendations from the epidemiology and quality of life working group of IOF. Osteoporos Int 2013; 24 (11): 2763–2764 10.1007/s00198-013-2413-7 PMC5096926 23884436

[8] Javaid MK, Sami A, Lems W (2020). A patient-level key performance indicator set to measure the effectiveness of fracture liaison services and guide quality improvement: a position paper of the IOF capture the fracture working group, National Osteoporosis Foundation and fragility fracture network. Osteoporos Int.

[9] Schousboe JT (2016). Epidemiology of vertebral fractures. J Clin Densitom.

[10] Chen W, Simpson JM, March LM, Blyth FM, Bliuc D, Tran T, Nguyen TV, Eisman JA, Center JR (2018). Comorbidities only account for a small proportion of excess mortality after fracture: a record linkage study of individual fracture types. J Bone Miner Res.

[11] Benzinger P, Riem S, Bauer J, Jaensch A, Becker C, Büchele G, Rapp K (2019). Risk of institutionalization following fragility fractures in older people. Osteoporos Int.

[12] Viswanathan M, Reddy S, Berkman N, Cullen K, Middleton JC, Nicholson WK, Kahwati LC (2018). Screening to Prevent Osteoporotic Fractures. Updated Evidence Report and Systematic Review for the US Preventive Services Task Force. Rockville (MD): Agency for Healthcare Research and Quality (US); 2018 Jun. Report no.: 15-05226-EF-1. JAMA.

[13] Hirsch JA, Beall DP, Chambers MR, Andreshak TG, Brook AL, Bruel BM, Deen HG, Gerszten PC, Kreiner DS, Sansur CA, Tutton SM, van der Meer P, Stoevelaar HJ (2018). Management of vertebral fragility fractures: a clinical care pathway developed by a multispecialty panel using the RAND/UCLA Appropriateness Method. Spine J.

[14] Pizzato S, Trevisan C, Lucato P, Girotti G, Mazzochin M, Zanforlini BM, Bano G, Piovesan F, Bertocco A, Zoccarato F, Dianin M, Manzato E, Sergi G (2018). Identification of asymptomatic frailty vertebral fractures in post-menopausal women. Bone..

[15] Gehlbach SH, Bigelow C, Heimisdottir M, May S, Walker M, Kirkwood JR (2000). Recognition of vertebral fracture in a clinical setting. Osteoporos Int.

[16] Delmas PD, van de Langerijt L, Watts NB, Eastell R, Genant H, Grauer A, Cahall DL, IMPACT Study Group (2005). Underdiagnosis of vertebral fractures is a worldwide problem: the IMPACT study. J Bone Miner Res.

[17] Fink HA, Milavetz DL, Palermo L, Nevitt MC, Cauley JA, Genant HK, Black DM, Ensrud KE, Fracture Intervention Trial Research Group (2005). What proportion of incident radiographic vertebral deformities is clinically diagnosed and vice versa?. J Bone Miner Res.

[18] Ensrud KE, Blackwell TL, Fink HA, Zhang J, Cauley JA, Cawthon PM, Black DM, Bauer DC, Curtis JR, Orwoll ES, Barrett-Connor E, Kado DM, Marshall LM, Shikany JM, Schousboe JT, for the Osteoporotic Fractures in Men (MrOS) Research Group (2016). Osteoporotic fractures in men (MrOS) research group. What proportion of incident radiographic vertebral fractures in older men is clinically diagnosed and vice versa: a prospective study. J Bone Miner Res.

[19] Mitchell RM, Jewell P, Javaid MK, McKean D, Ostlere SJ (2017). Reporting of vertebral fragility fractures: can radiologists help reduce the number of hip fractures. Arch Osteoporos.

[20] McCabe E, Ibrahim A, Singh R, Kelly M, Armstrong C, Heaney F, Bergin D, McCabe JP, Carey JJ (2020). A systematic review of the Irish osteoporotic vertebral fracture literature. Arch Osteoporos.

[21] Beringer T, Heyburn G, Finch M, McNally C, McQuilken M, Duncan M, Dixon T (2006). Prevalence of vitamin D inadequacy in Belfast following fragility fracture. Curr Med Res Opin.

[22] Wright S, Beringer T, Taggart H, Keegan D, Kelly J, Whithead E, McKane R, McNally C, McQuilken M, Finch M (2007). A study of male patients with forearm fracture in Northern Ireland. Clin Rheumatol.

[23] Howlett DC, Drinkwater KJ, Mahmood N, Illes J, Griffin J, Javaid K (2020) Radiology reporting of osteoporotic vertebral fragility fractures on computed tomography studies: results of a UK national audit [published online ahead of print, 2020 May 20]. Eur Radiol. 10.1007/s00330-020-06845-2 10.1007/s00330-020-06845-2 32435926

[24] Barton DW, Behrend CJ, Carmouche JJ (2017) Osteoporosis management following vertebral fracture remains inadequate when secondary fracture prevention programs are not implemented. NASS 32nd Annual Meeting Proceedings / The Spine Journal 17. S41–S88

[25] Fracture Liaison Service Database (FLS-DB): https://www.rcplondon.ac.uk/projects/fracture-liaison-service-database-fls-db, last accessed 01/04/2020

